# Risk factors for residual low back pain at twelve months after low-temperature plasma radiofrequency ablation in lumbar disc herniation: a retrospective cohort study

**DOI:** 10.1007/s00264-026-06928-7

**Published:** 2026-06-27

**Authors:** Yingying Lou, Song Zhou, Yonggan Ying

**Affiliations:** Ningbo Medical Center Lihuili Hospital, Ningbo, China

**Keywords:** Lumbar disc herniation, Low-temperature plasma radiofrequency ablation, Residual low back pain, Risk factors, Pfirrmann grade

## Abstract

**Objectives:**

To investigate the incidence of rLBP and factors independently associated with residual low back pain (rLBP) at 12 months following low-temperature plasma radiofrequency ablation (LTP-RFA) in patients with lumbar disc herniation (LDH).

**Methods:**

This retrospective cohort study included patients with symptomatic LDH who were admitted in Ningbo Medical Center Lihuili Hospital and underwent LTP-RFA between January 2018 and January 2024. Baseline, clinical, and radiological data were collected. Pain and functional outcomes were assessed using the Visual Analogue Scale (VAS), Oswestry Disability Index (ODI), and Japanese Orthopaedic Association (JOA) score. rLBP was defined as the persistence or recurrence of low back pain with a VAS score ≥ 2 for a duration of at least six months after LTP-RFA. Univariate and multivariate logistic regression analyses were performed to identify factors independently associated with rLBP.

**Results:**

A total of 281 patients were included. At 12 months, 32.03% (90/281) of patients developed rLBP. Overall, significant improvements were observed in VAS, ODI, and JOA scores after surgery (all *P* < 0.001). Multivariate analysis identified higher occupational physical workload, advanced disc degeneration (Pfirrmann grade III–IV), and non-central herniation types as factors independently associated with rLBP. Heavy physical labour (OR = 15.951, *P* < 0.001) and higher Pfirrmann grades (grade IV: OR = 13.086, *P* < 0.001) were associated with higher odds of rLBP. Compared with central herniation, extreme lateral herniation showed the highest odds (OR = 8.146, *P* < 0.001).

**Conclusion:**

rLBP was a common outcome after LTP-RFA. Occupational physical workload, herniation type, and Pfirrmann grade were associated with postoperative rLBP. Preoperative risk stratification and targeted postoperative management may assist in perioperative risk assessment and patient counseling.

## Introduction

Lumbar disc herniation (LDH) is one of the most common degenerative spinal disorders, imposing a substantial clinical and socioeconomic burden worldwide. The annual incidence of LDH with radiculopathy in the general population ranges from 1–5 per 1,000 persons, with higher rates observed in occupational populations and those exposed to heavy physical labour [1]. LDH is a leading cause of low back pain (LBP), leg pain, and work-related disability, significantly impairing patients' quality of life and productivity [2]. Approximately 70–90% of patients improve with conservative treatment, whereas about 10% of patients with symptomatic LDH ultimately require surgical intervention due to persistent symptoms or progressive neurological deficits [3, 4].

Among the various minimally invasive surgical techniques, low-temperature plasma radiofrequency ablation (LTP-RFA) has emerged as a promising treatment modality for LDH [5]. This technique uses a low-temperature plasma field (typically operates between 40–70 °C but can vary depending on energy settings and application) to ablate a small portion of the nucleus pulposus, thereby reducing intradiscal pressure and achieving indirect decompression of the affected nerve root [6]. Compared with conventional open discectomy, LTP-RFA has been associated with smaller incisions, less soft-tissue trauma, reduced blood loss, shorter hospital stay, and faster postoperative recovery [7, 8]. Previous studies [9] have demonstrated that LTP-RFA can effectively alleviate radicular pain and improve functional outcomes in selected LDH patients.

Despite favourable symptom relief after LTP-RFA, a subset of patients continues to experience persistent or recurrent LBP (rLBP) following the procedure [7]. LBP represents a common and clinically challenging problem, as it may compromise patients' functional recovery and overall satisfaction with surgical outcomes. Long-term follow-up studies [10, 11] have reported that the prevalence of persistent LBP after lumbar disc herniation surgery has been reported to range from 20 to 45% depending on the diagnostic criteria and follow-up duration, markedly higher than that in the general population. Furthermore, patients with rLBP often exhibit worse postoperative functional scores, including higher Oswestry Disability Index (ODI) and lower Japanese Orthopaedic Association (JOA) scores, underscoring the substantial impact of rLBP on patients' daily activities and quality of life [12].

Although risk factors for rLBP have been extensively studied following open discectomy and percutaneous endoscopic lumbar discectomy (PELD), evidence specific to LTP-RFA are lacking [11]. Existing LTP-RFA literature [12] has predominantly focused on short-term pain relief efficacy, with limited attention to predictors of unfavourable long-term outcomes. Given the increasing adoption of LTP-RFA in clinical practice, a comprehensive understanding of the factors associated with rLBP is essential for improving patient selection and perioperative decision-making. Therefore, the present retrospective cohort study was aimed to systematically investigate the potential factors associated with rLBP at 12 months following LTP-RFA in patients with LDH.

## Materials and methods

### Study design and patients

This retrospective cohort study was conducted at the Department of Pain Medicine, Ningbo Medical Center Lihuili Hospital, China. Consecutive patients who underwent LTP-RFA for symptomatic LDH between January 2018 and January 2024 were screened for eligibility. The study protocol was approved by the Institutional Ethics Committee of Ningbo Medical Center Lihuili Hospital (NO. KY2024SL483-01), and the requirement for informed consent was waived due to the retrospective nature of the study.

The inclusion criteria were as follows: (1) age 20–65 years; (2) diagnosis of single- or multi-level LDH confirmed by lumbar magnetic resonance imaging (MRI) and computed tomography (CT); (3) presence of radicular pain or neurological deficits corresponding to the affected segment; (4) failure of conservative treatment for at least three months; (5) complete preoperative and postoperative clinical and imaging data available.

The exclusion criteria included: (1) previous lumbar spine surgery; (2) spinal tumors, infections, or fractures; (3) uncontrolled psychiatric disorders or cognitive impairment; (4) change of treatment plan after enrollment; (5) loss to follow-up; and (6) other conditions that could interfere with outcome assessment.

### Data collection

Baseline characteristics were extracted from the electronic medical records, including age, sex, body mass index (BMI), smoking history, alcohol consumption history, and comorbidities (hypertension, diabetes mellitus). The occupational physical workload classification was determined according to Liu et al*.* [13]. Clinical characteristics encompassed the direction (unilateral, bilateral, or central), location (leg, hip, or lumbar), and nature (pain, numbness, or muscle weakness) of symptoms, as well as the duration of symptoms prior to surgery.

### Assessment of pain and functional scores

Pain and functional scores were assessed using the Visual Analogue Scale (VAS) for pain [14], the JOA score [15], and the ODI [16]: (1) VAS: The VAS is a 10-point scale ranging from 0 (no pain) to 10 (worst imaginable pain); (2) ODI: The ODI is a validated questionnaire consisting of ten items covering pain intensity and functional activities, including personal care, lifting, walking, sitting, standing, sleeping, sexual activity, social life, and traveling, with a total score ranging from 0 to 100, where higher scores indicate greater disability; (3) JOA: The JOA score (maximum 29 points) assesses lumbar function across four domains: subjective symptoms, activities of daily living, clinical signs, and bladder function, with lower scores indicating more severe impairment. All scores were recorded preoperatively and at the final follow-up (12 months postoperatively).

### Imaging diagnostic criteria for LDH

**Fig. 1:**
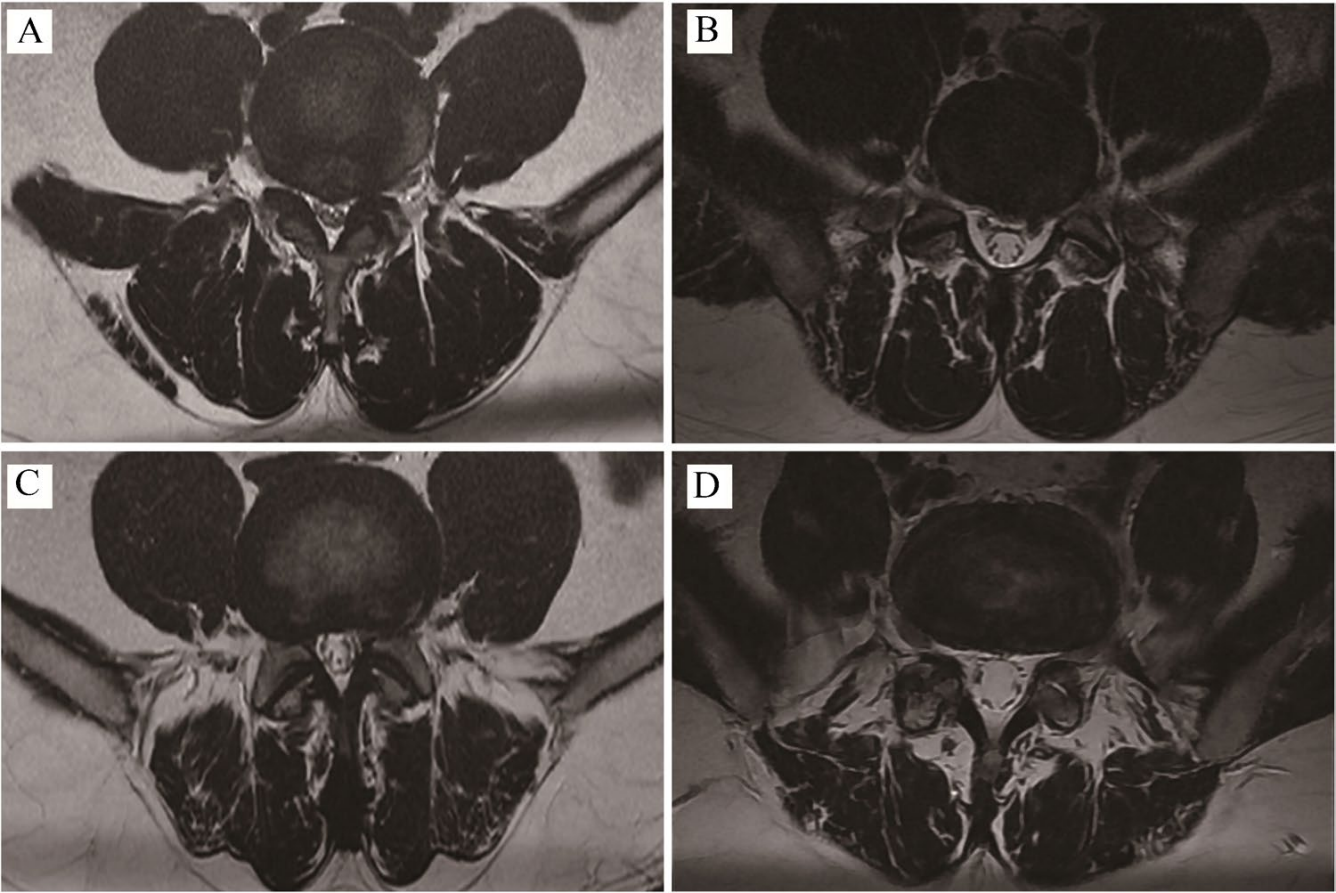
MRI classification of disc herniation. (**A**) Central type; (**B**) Lateral recess type; (**C**) Foraminal type; (**D**) Extreme lateral type

**Fig. 2:**
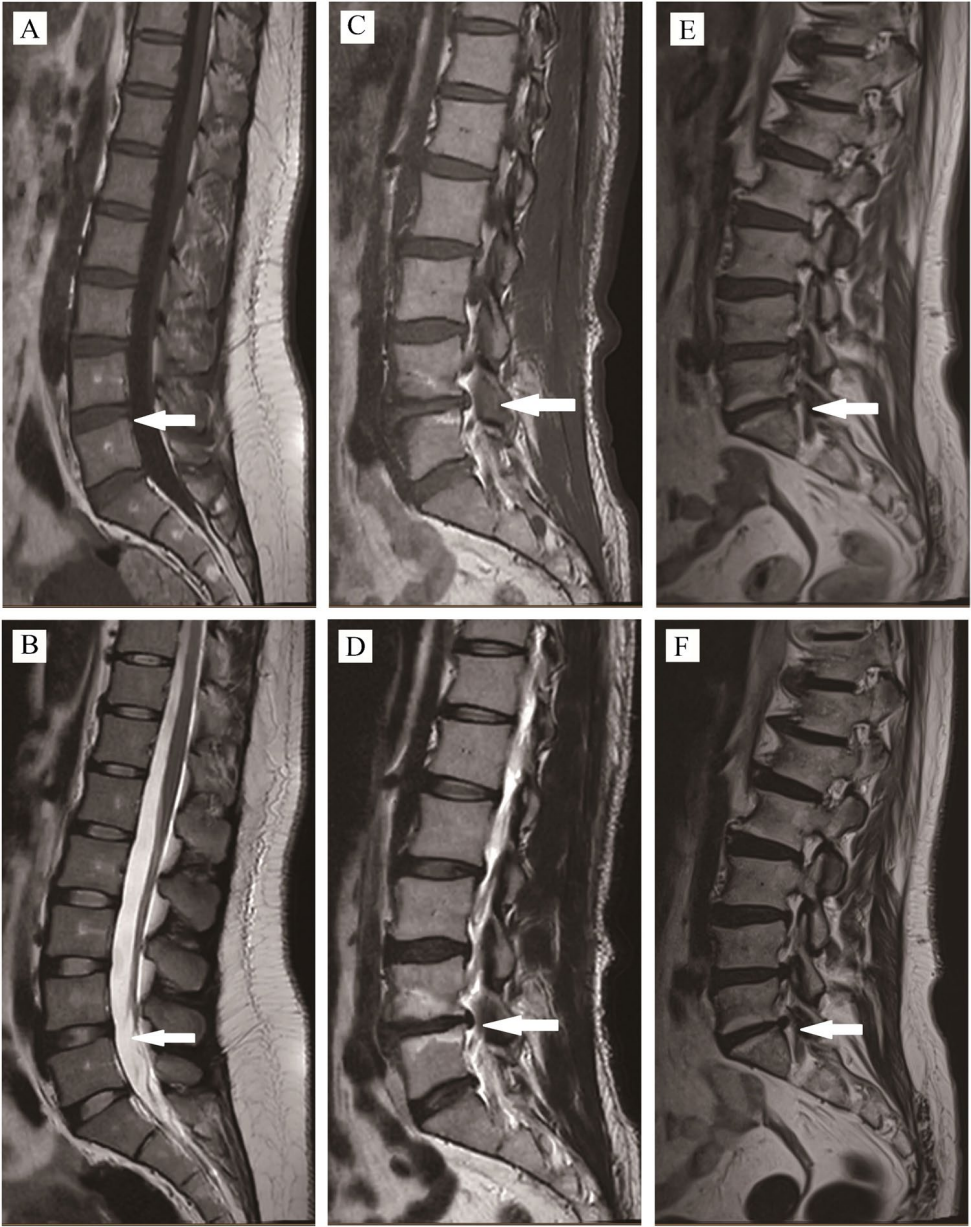
Appearance of Modic changes in intervertebral discs. (**A**, **B**) Normal discs; (**C**, **D**) Type I, Hypointensity onT1-w and hyperintensity onT2-w. (**E**, **F**) Type II, hyperintensity on T1-w and T2-w (White arrow). MRI, Magnetic resonance imaging; -w, weighted imaging

**Fig. 3:**
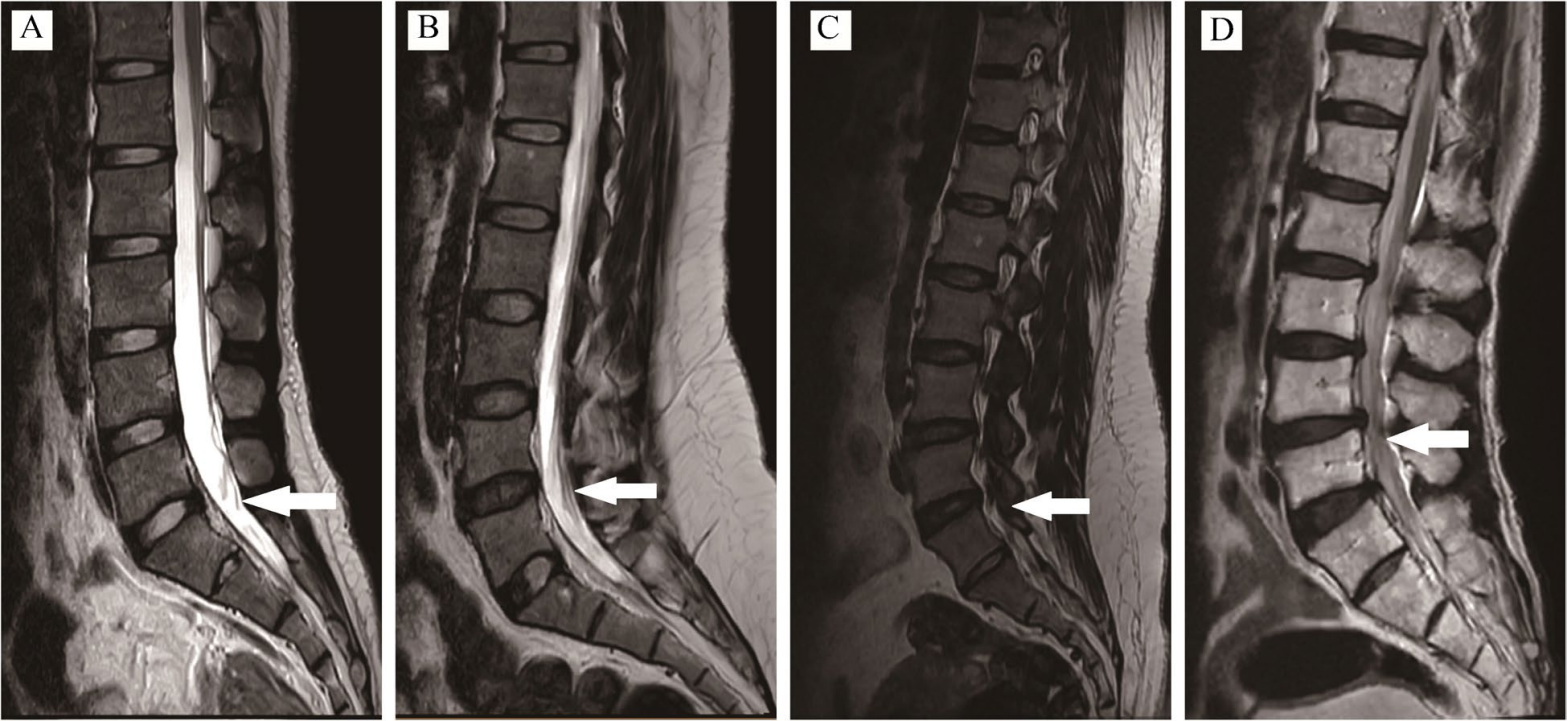
Pfirrmann grading of intervertebral discs on MRI. (**A**) Grade I; (**B**) Grade II; (**C**) Grade III; (**D**) Grade IV (White arrow)

**Fig. 4:**
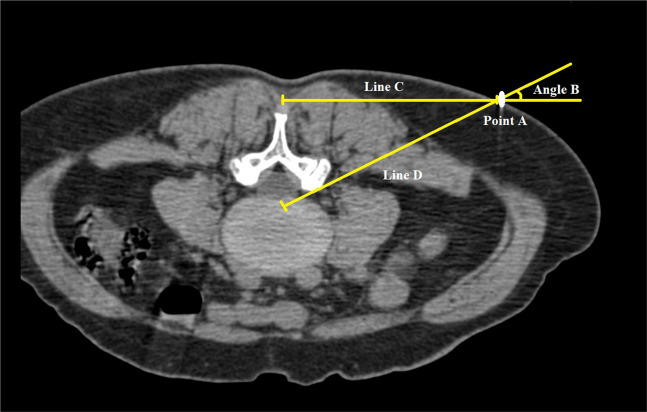
Radiographic evaluation of the puncture trajectory under CT guidance. Point A: Entry point; Angle B: Puncture angle; Line C: Lateral distance; Line D: Puncture trajectory

**Fig. 5:**
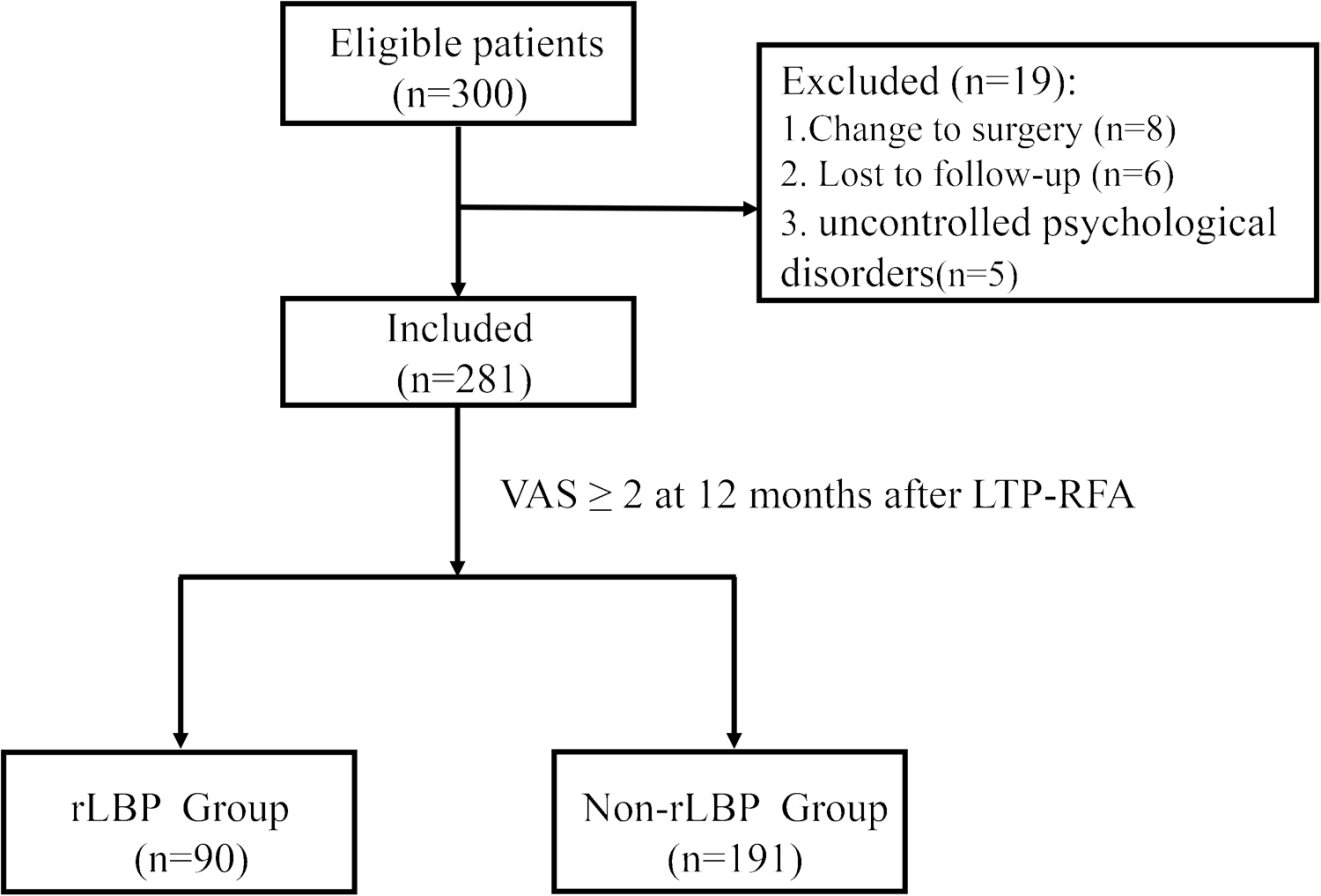
Flowchart of the study design

All patients underwent preoperative lumbar MRI and CT examinations. The following imaging parameters were evaluated: (1) Affected segment and number of involved discs: The specific intervertebral levels (L1/2, L2/3, L3/4, L4/5, L5/S1) and the number of herniated discs were recorded; (2) Type of herniation [17]: Based on the anatomical location of the herniated disc relative to the neural structures, herniations were classified as central, lateral recess, foraminal, or extreme lateral types (Fig. 1); (3) Modic changes [18]: Vertebral endplate signal changes on MRI were classified as Type I (hypointense on T1-weighted imaging and hyperintense on T2-weighted imaging), Type II (hyperintense on both T1- and T2-weighted imaging (Fig. 2); (4) Pfirrmann grade [19]: Intervertebral disc degeneration was graded on sagittal T2-weighted MRI using the Pfirrmann classification system (Grade I–IV), with higher grades indicating more severe degeneration (Fig. 3); (5) Lumbar spinal stenosis: The presence or absence of central canal or foraminal stenosis was documented.

### Surgical procedure of LTP-RFA

All LTP-RFA procedures were performed in a CT operating room under strict sterile conditions by a unified surgical team with extensive experience in minimally invasive spinal interventions. Patients were placed in the prone position, and the responsible intervertebral disc was identified under CT guidance. The target disc level was marked, and the puncture entry point (Point A) was determined according to the planned puncture angle (Angle B, defined as the angle between Line C and Line D), width (Line C, the horizontal line between the body surface marker and the spinous process), and puncture path (Line D, the distance from the body surface marker to the herniation site) (Fig. 4).

After routine skin preparation and draping, local anesthesia was achieved using 1% lidocaine with layered infiltration. A 19-gauge puncture needle with an internal mandrel was advanced via a posterolateral approach through the facet joint into Kambin’s triangle and positioned within the target disc under CT guidance. Once CT confirmed that the needle tip had entered the annulus fibrosus and reached the herniated target, the stylet was removed and a low-temperature plasma radiofrequency ablation probe (SM-D380C; Gaotong, Xi’an, China) was inserted. Ablation was performed using the ablation mode for 10–15 s followed by coagulation mode for 5–10 s, repeated for three cycles. During the procedure, the operation was immediately halted if the puncture channel approached the nerve root or if patients experienced lower-limb discomfort. The plasma-generated energy field ablated and vaporized a portion of the herniated nucleus pulposus, thereby reducing intradiscal pressure and achieving indirect nerve root decompression while preserving surrounding annulus fibrosus and neural structures. Surgical parameters, including energy level and ablation duration, were standardized according to the manufacturer’s protocol and adjusted based on the herniation type and volume.

Postoperatively, patients were monitored in the ward for potential complications. Routine wound care and analgesics were provided as needed, and gradual mobilization was encouraged. Patients were instructed to wear lumbar support for one week, avoid heavy physical activity, and maintain proper posture during early recovery. Follow-up evaluations were conducted at 12 months postoperatively through outpatient visits or telephone interviews.

### Grouping

Patients were divided into two groups based on their rLBP status at the 12-month follow-up. rLBP was defined as the persistence or recurrence of LBP with a VAS score ≥ 2 for a duration of at least six months after LTP-RFA [20, 21]. Patients meeting these criteria were assigned to the rLBP group, while those with VAS scores < 2 or without persistent LBP constituted the non-rLBP group.

### Statistical analysis

Statistical analyses were performed using SPSS version 26.0 (IBM Corp., Armonk, NY, USA). Continuous variables were tested for normality using the Shapiro–Wilk test and were found to be non-normally distributed. Therefore, data were presented as median (interquartile range, IQR). Categorical variables were presented as frequencies and percentages. Comparisons between the rLBP and non-rLBP groups were conducted using Mann–Whitney U test for continuous variables, and the chi-squared test or Fisher's exact test for categorical variables, as appropriate. Preoperative and postoperative functional scores within each group were compared using Wilcoxon signed ranks test. Variables with statistical significance (*P* < 0.05) in the univariate analysis were further entered into a multivariate logistic regression model to identify factors independently associated with rLBP. Multicollinearity among variables was assessed prior to model construction, and highly correlated variables were excluded to avoid collinearity bias. Odds ratios (OR) with 95% confidence intervals (CI) were calculated. A two-sided *P*-value < 0.05 was considered statistically significant.

## Results

### Patient characteristics

A total of 300 patients were initially screened in this study. According to the predefined exclusion criteria, 19 patients were excluded, including eight due to changes in treatment strategy, 6 lost to follow-up, and five with uncontrolled psychological disorders. Ultimately, 281 patients met the inclusion criteria and were included in the final analysis (Fig. 5).

Regarding baseline characteristics, the median age was 44.00 years (IQR: 36.00–52.00), and 45.20% of the patients were male. The median body mass index (BMI) was 21.67 kg/m^2^ (IQR: 19.11–24.11). The prevalence of hypertension and diabetes was 32.74% and 33.10%, respectively. A history of smoking was reported in 47.69% of patients, while 53.02% had a history of alcohol consumption. Moderate-intensity physical labor was reported in 52.31% of patients (Table 1).

**Table 1:** Comparison of baseline, clinical, and imaging characteristics between the rLBP and non-rLBP groups

Variables	Total (*n* = 281)	Non-rLBP group (*n* = 191)	rLBP group (*n* = 90)	Statistics	P
**Baseline characteristics**					
Age, median (IQR)	44.00 (36.00, 52.00)	45.00 (36.00,53.00)	43.00 (35.00,52.00)	−0.555	0.579*
Sex, *n* (%)				1,234	0.267#
Male	127 (45.20)	82(42.93)	45(50.00)		
Female	154 (54.80)	109(57.07)	45(50.00)		
BMI, median (IQR)	21.67 (19.11, 24.11)	21.78 (19.15,24.44)	21.64 (18.94,23.52)	−0.631	0.528*
Hypertension history, *n* (%)				0.016	0.899#
NO	189 (67.26)	128 (67.02)	61 (67.78)		
YES	92 (32.74)	63 (32.98)	29 (32.22)		
Diabetes history, *n* (%)				0.003	0.954#
NO	188 (66.90)	128 (67.02)	60 (66.67)		
YES	93 (33.10)	63 (32.98)	30 (33.33)		
Smoking history, *n* (%)				0.055	0.814#
NO	147 (52.31)	99(51.83)	48(53.33)		
YES	134 (47.69)	92(48.17)	42(46.67)		
Drinking history, *n* (%)				0.705	0.401#
NO	132 (46.98)	93(48.69)	39(43.33)		
YES	149 (53.02)	98(51.31)	51(56.67)		
Occupational physical workload				16.989	< 0.001#
Light	53 (18.86)	43(22.51)	10(11.11)		
Moderate	147 (52.31)	107(56.02)	40(44.44)		
Heavy	81 (28.83)	41(21.47)	40(44.44)		
**Clinical characteristics**					
Direction of symptoms, *n* (%)				0.218	0.897 #
Unilateral	110 (39.15)	73(38.22)	37(41.11)		
Bbilateral	64 (22.78)	44(23.04)	20(22.22)		
Central	107 (38.08)	74(38.74)	33(36.67)		
Location of symptoms, *n* (%)				0.222	0.895#
Leg	61 (21.71)	40(20.94)	21(23.33)		
Hip	113 (40.21)	78(40.84)	35(38.89)		
Lumbar	107 (38.08)	73(38.22)	34(37.78)		
Nature of symptoms, *n* (%)				1.681	0.432#
Pain	180 (64.06)	118(61.78)	62(68.89)		
Numbness	66 (23.49)	49(25.65)	17(18.89)		
Muscle weakness	35 (12.46)	24(12.57)	11(12.22)		
Duration of symptom, *n* (%)				2.022	0.155#
≤ 3 months	136 (48.40)	98 (51.31)	38 (42.22)		
> 3 months	145 (51.60)	93 (48.69)	52 (57.78)		
**Imaging characteristics**					
Modic change, n (%)				41.993	< 0.001#
NO	154 (54.80)	129 (67.54)	25 (27.78)		
Modic I	77 (27.40)	42 (25.13)	35 (23.33)		
Modic II	50 (17.79)	20 (20.94)	30 (36.67)		
Lumbar spinal stenosis, *n* (%)				0.060	0.807#
NO	221 (78.65)	151(79.06)	70(77.78)		
YES	60 (21.35)	40(20.94)	20(22.22)		
Pfirrmann grade, *n* (%)				57.842	< 0.001#
Type I	133 (47.33)	119 (62.30)	14 (15.56)		
Type II	42 (14.95)	23 (12.04)	19 (21.11)		
Type III	47 (16.73)	26 (13.61)	21 (23.33)		
Type IV	59 (21.00)	23 (12.04)	36 (40.00)		
Segment, *n* (%)				15.672	0.002@
L1/2	2 (0.71)	2(1.05)	0(0)		
L2/3	12 (4.27)	6(3.14)	6(6.67)		
L3/4	29(10.32)	15(7.85)	14(15.56)		
L4/5	128 (45.55)	101(52.88)	27(30)		
L5/S1	110 (39.15)	67(35.08)	43(47.78)		
Number of damaged discs, *n* (%)				2.720	0.455@
1	118 (41.99)	80(41.88)	38(42.22)		
2	147 (52.31)	98(51.31)	49(54.44)		
3	10 (3.56)	7(3.66)	3(3.33)		
4	6 (2.14)	6(3.14)	0(0)		
Type of Herniation, *n* (%)				30.323	< 0.001#
Central	110 (39.15)	92 (48.17)	18 (20.00)		
Lateral recess	69 (24.56)	48 (25.13)	21 (23.33)		
Foraminal	73 (25.98)	40 (20.94)	33 (36.67)		
Extreme Latera	29 (10.32)	11 (5.76)	18 (20.00)		

*Mann–Whitney U Test; #Chi-squared Test; @ Fisher's Exact Test

In terms of clinical characteristics, unilateral symptoms were the most common (39.15%). Symptoms were primarily distributed in the hip (40.21%) and lower back (38.08%). Pain was the predominant clinical manifestation (64.06%). The duration of symptoms exceeded three months in 51.60% of patients (Table 1).

Regarding imaging findings, 45.20% of patients exhibited Modic changes (Modic type I: 27.40%; Modic type II: 17.79%), and 21.35% had concomitant lumbar spinal stenosis. Pfirrmann grade I was the most common (47.33%). The most frequently involved level was L4/5 (45.55%), followed by L5/S1 (39.15%). Most patients had involvement of two intervertebral discs (52.31%). Central-type disc herniation was the most common subtype (39.15%) (Table 1).

### Comparison of preoperative and postoperative pain and functional scores

As shown in Table 2, significant postoperative improvements were observed across all outcome measures in the entire cohort of 281 patients. The median VAS score decreased from 4.00 preoperatively to 1.00 postoperatively (Z =  − 14.560, *P* < 0.001), the median ODI score decreased from 55.00 to 15.00 (Z =  − 14.412, *P* < 0.001), and the median JOA score increased from 14.00 to 22.00 (Z =  − 14.524, *P* < 0.001).

**Table 2:** Comparison of preoperative and postoperative VAS, ODI, and JOA scores

Scores	Groups	Pre-, median (IQR)	Post-, median (IQR)	Z	P*
**VAS score**	Overall (*n* = 281)	4.00 (4.00, 5.00)	1.00 (0.00, 2.00)	−14.560	< 0.001
	Non-rLBP group (*n* = 191)	4.00 (3.00,5.00)	1.00 (0.00,1.00)	−12.115	< 0.001
	rLBP group (*n* = 90)	4.00 (4.00,5.00)	2.00 (2.00,3.00)	−8.370	< 0.001
**JOA score**	Overall (*n* = 281)	14.00 (12.00, 15.00)	22.00 (20.00, 23.00)	−14.524	< 0.001
	Non-rLBP group (*n* = 191)	14.00 (12.00,15.00)	23.00 (21.00,24.00)	−11.937	< 0.001
	rLBP group (*n* = 90)	15.00 (12.00,15.00)	19.00 (18.00,20.25)	−8.091	< 0.001
**ODI score**	Overall (*n* = 281)	55.00 (45.00,64.00)	15.00 (12.00, 24.00)	−14.412	< 0.001
	Non-rLBP group (*n* = 191)	56.00 (45.00,65.00)	14.00 (12.00,17.00)	−11.986	< 0.001
	rLBP group (*n* = 90)	55.00 (43.00,62.00)	26.00 (19.00,30.00)	−8.201	< 0.001

*Wilcoxon signed ranks test

During the 12-month follow-up period, no patients required reintervention after surgery. At the 12-month follow-up, 90 (32.03%) and 191 (67.97%) patients were classified into the rLBP and non-rLBP groups, respectively (Fig. 1). Subgroup analyses further demonstrated that both the non-rLBP and rLBP groups showed significant postoperative improvements in VAS, ODI, or JOA scores (all *P* < 0.001, Table 2).

Intergroup comparisons revealed no significant differences in preoperative VAS, ODI, or JOA scores between the two groups (all *P* > 0.05, Table 3), suggesting similar preoperative symptom severity and functional status. However, at the final follow-up, compared with the rLBP group, the non-rLBP group showed significantly lower VAS scores (median 1.00 vs. 2.00, Z =  − 14.238, *P* < 0.001) and ODI scores (median 14.00 vs. 26.00, Z =  − 9.354, *P* < 0.001), as well as higher JOA scores (median 23.00 vs. 19.00, Z =  − 8.861, *P* < 0.001).

**Table 3:** Comparison of VAS, ODI, and JOA scores between the non-rLBP and rLBP groups

Scores	Non-rLBP group (*n* = 191)	rLBP group (*n* = 90)	Z	P*
VAS score, median (IQR)				
Pre-	4.00 (3.00,5.00)	4.00 (4.00,5.00)	−1.907	0.057
Post-	1.00 (0.00,1.00)	2.00 (2.00,3.00)	−14.238	< 0.001
ODI score, median (IQR)				
Pre-	56.00 (45.00,65.00)	55.00(43.00,62.00)	−1.659	0.097
Post-	14.00 (12.00,17.00)	26.00(19.00,30.00)	−9.354	< 0.001
JOA score, median (IQR)				
Pre-	14.00 (12.00,15.00)	15.00(12.00,15.00)	−0.132	0.895
Post-	23.00 (21.00,24.00)	19.00(18.00,20.25)	−8.861	< 0.001

*Mann–Whitney U test

### Univariate analysis of risk factors associated with rLBP

To explore potential risk factors for rLBP, univariate analyses were performed comparing the rLBP and non-rLBP groups. Results showed that the following variables were significantly associated with rLBP: occupational physical workload (χ^2^ = 16.989, *P* < 0.001), Modic changes (χ^2^ = 41.993, *P* < 0.001), Pfirrmann grade (χ^2^ = 57.842, *P* < 0.001), surgical segment (χ^2^ = 15.672, *P* = 0.002), and type of disc herniation (χ^2^ = 30.323, *P* < 0.001). No significant associations were observed for age, sex, BMI, hypertension history, diabetes history, smoking history, drinking history, direction, location, nature, and duration of symptoms, lumbar spinal stenosis, and number of damaged discs) (all *P* > 0.05) (Table 1).

### Multivariate logistic regression analysis of risk factors associated with rLBP

Variables that were significant in the univariate analysis (occupational physical workload, Modic changes, surgical segment, Pfirrmann grade, and type of herniation) were further included in a multivariate logistic regression model. Significant associations were observed between Modic changes and type of herniation (χ^2^ = 15.692, *P* = 0.016), as well as between surgical segment and type of herniation (χ^2^ = 115.441, *P* < 0.001), suggesting potential collinearity among these variables. Therefore, in the multivariate logistic regression analysis**,** occupational physical workload, Pfirrmann grade, and type of herniation were ultimately included as independent variables, with postoperative rLBP as the dependent variable.

As shown in Table 4, results of multivariate logistic regression analysis showed that extreme lateral herniation, higher Pfirrmann grades (III–IV), and heavy physical workload were significantly associated with higher odds of rLBP. Specifically: (1) Occupational physical workload: Compared to patients with light physical workload, those with moderate physical workload were independently associated with higher odds of rLBP (OR = 4.345, 95% CI: 1.470–12.848, *P* = 0.008), while those with heavy physical workload showed the strongest association (OR = 15.951, 95% CI: 5.158–49.324, *P* < 0.001); (2) Type of herniation: Compared to patients with central herniation, those with lateral recess herniation (OR = 2.834, 95% CI: 1.185–6.780, *P* = 0.019), foraminal herniation (OR = 3.327, 95% CI: 1.487–7.443, *P* = 0.003), and extreme lateral herniation (OR = 8.146, 95% CI: 2.684–24.727, *P* < 0.001) were associated with progressively higher odds; (3) Pfirrmann grade: compared to patients with Pfirrmann grade I, those with Pfirrmann grade II (OR = 6.540, 95% CI: 2.609–16.396, *P* < 0.001), Pfirrmann grade III (OR = 7.561, 95% CI: 3.085–18.532, *P* < 0.001), and Pfirrmann grade IV (OR = 13.086, 95% CI: 5.426–31.560, P < 0.001) were associated with progressively greater odds of rLBP.

**Table 4:** Multivariate logistic regression analysis of risk factors associated with postoperative rLBP

Variables	OR (95% CI)	P
**Occupational physical workload,** *n* (%)		
Light	1	
Moderate	4.345 (1.47–12.848)	0.008
Heavy	15.951 (5.158–49.324)	< 0.001
**Pfirrmann grade,** *n* (%)		
Type I	1	
Type II	6.540 (2.609–16.396)	< 0.001
Type III	7.561 (3.085–18.532)	< 0.001
Type IV	13.086 (5.426–31.56)	< 0.001
**Type of Herniation,** *n* (%)		
Central	1	
Lateral recess	2.834 (1.185–6.78)	0.019
Foraminal	3.327 (1.487–7.443)	0.003
Extreme Latera	8.146 (2.684–24.727)	< 0.001

## Discussion

The present retrospective cohort study investigated the incidence of rLBP and factors associated with rLBP following LTP-RFA in patients with LDH. Of the 281 patients included in the final analysis, 90 (32.0%) developed rLBP at 12 months postoperatively. Multivariate logistic regression analysis identified three factors independently associated with rLBP: occupational physical workload, Pfirrmann grade, and type of herniation. Specifically, extreme lateral herniation, advanced disc degeneration (Pfirrmann Grade III–IV), and heavy physical labour were significantly associated with higher odds of rLBP. These findings may improve understanding of factors associated with rLBP after LTP-RFA and may assist in perioperative patient counseling and risk stratification.

Our study demonstrated a possible graded association between occupational physical workload and the risk of rLBP. Compared with light physical workload, moderate and heavy physical workloads were associated with 4.345-fold and 15.951-fold higher odds of rLBP, respectively. Our findings were consistent with accumulating evidence indicating that higher occupational physical workload is associated with increased odds of LBP in a dose–response manner [22, 23]. Previous studies [24, 25] have suggested that repetitive mechanical loading, prolonged bending, and lifting may contribute to accelerated degeneration and inflammatory responses. In the context of LTP-RFA, although partial ablation of the nucleus pulposus is achieved, underlying disc degeneration and biomechanical instability may persist. Therefore, patients engaged in heavy physical workload may be exposed to sustained mechanical stress, which may hinder tissue recovery, increase the risk of recurrent microtrauma, and contribute to rLBP [26]. These findings suggest the potential value of considering occupational factors in preoperative evaluation and postoperative activity modification, as well as ergonomic interventions.

In this study, disc degeneration severity, as quantified by the Pfirrmann grade, was independently associated with rLBP. Higher Pfirrmann grades were associated with progressively higher odds of rLBP, with Grade IV degeneration corresponding to a 13.086-fold higher odds compared with Grade I. This finding was consistent with previous evidence suggesting that advanced disc degeneration is associated with chronic LBP [27]. The Pfirrmann grading system reflects the progressive loss of nucleus pulposus hydration, structural disorganization of the annulus fibrosus, and reduction in disc height, all of which may contribute to abnormal segmental biomechanics and load distribution [28]. In patients with severe degeneration, LTP-RFA primarily addresses the herniated component but does not reverse the underlying degenerative changes. Biomechanical studies [29, 30] have suggested that the compromised disc structure may continue to transmit abnormal mechanical loads to the vertebral endplates and facet joints, potentially perpetuating a cycle of microtrauma, inflammation, and pain. Previous studies [31, 32] have similarly reported that more advanced disc degeneration, as reflected by higher Pfirrmann grades, may be associated with less favourable clinical outcomes following minimally invasive disc procedures. Therefore, Pfirrmann grade may be considered in preoperative counseling regarding the expected durability of pain relief and the potential need for adjunctive treatments or future fusion surgery.

The anatomical type of disc herniation was independently associated with rLBP in our cohort. Compared with central herniation, lateral recess, foraminal, and extreme lateral herniations were associated with progressively higher odds of rLBP, with extreme lateral herniation demonstrating the strongest association [33]. This may be explained by several biomechanical and pathophysiological factors. Foraminal and extraforaminal herniations more frequently involve the exiting nerve root and dorsal root ganglion (DRG). Anatomically, the DRG in the lower lumbar spine is predominantly located within the intervertebral foramen, close to the rostral pedicle, making it particularly vulnerable to compression from lateral herniations [34]. Moreover, the anatomical constraints of the foraminal and extraforaminal regions may limit surgical access and reduce the extent of adequate decompression achievable with LTP-RFA, potentially increasing the likelihood of residual disc material and persistent neural irritation [35]. These findings suggest that closer postoperative monitoring may be considered in patients with lateral and extreme lateral herniations.

Although Modic changes and surgical level were significant in univariate analyses, they were excluded from the multivariable model to avoid multicollinearity with herniation type. Modic changes, reflecting vertebral endplate bone marrow alterations associated with inflammatory and degenerative processes, have been associated with chronic LBP in previous studies [36], consistent with the finding in our cohort. The predominance of L4/5 and L5/S1 levels may reflect the caudal gradient of biomechanical loading and disc degeneration [37], as these segments are subject to greater mobility and higher axial pressure [38]. Collectively, these factors may reflect overlapping biomechanical and degenerative mechanisms underlying herniation patterns [39, 40]. Notably, conventional factors such as age, BMI, smoking, and diabetes were not associated with rLBP in this study, suggesting that local biomechanical or procedure-related factors may play a relatively greater role within this cohort, although the contribution of systemic factors cannot be excluded.

This study has several limitations that should be acknowledged. First, the retrospective design inherently introduces the risk of selection bias and information bias, and causal inference cannot be established. Second, the single-centre design may limit the generalizability of our findings, as patient characteristics, surgical practices, and follow-up strategies may differ across institutions. Third, although the sample size was sufficient for the primary analysis, statistical power may have been limited for detecting associations involving infrequent exposures or modest effect sizes. Fourth, several potential confounders, such as psychosocial factors, postoperative rehabilitation adherence, and paraspinal muscle quality, were not systematically evaluated due to data unavailability, which may have introduced residual confounding. Fifth, the follow-up duration was limited to 12 months, and longer-term outcomes, such as recurrent disc herniation or the need for revision surgery, remain unknown. Sixth, although many patients had two affected discs, only the symptomatic "responsible segment" (determined by exam, localization, and imaging) was treated. Due to the small number of simultaneous two-disc surgeries, we could not meaningfully compare postoperative pain recurrence between single-disc and two-disc groups.

## Conclusion

In conclusion, rLBP occurred in 32% of patients at 12 months after LT-RFA for LDH in our cohort study. Higher occupational physical workload, advanced disc degeneration, and non-central, particularly extreme lateral herniation types, were independently associated with higher odds of rLBP. These findings suggest the potential value of integrating clinical and radiological parameters into risk stratification and patient counseling. Further prospective, multicenter studies are required to validate these observations and improve the robustness of risk assessment.

## Data Availability

No datasets were generated or analysed during the current study.
